# Perceived Stress of Quarantine and Isolation During COVID-19 Pandemic: A Global Survey

**DOI:** 10.3389/fpsyt.2021.656664

**Published:** 2021-05-25

**Authors:** TMGH-Global COVID-19 Collaborative, Nguyen Tien Huy

**Affiliations:** Author Affiliations: School of Tropical Medicine and Global Health, Nagasaki University, Nagasaki, Japan; University of Medicine and Pharmacy at Ho Chi Minh City, Ho Chi Minh City, Vietnam; Faculty of Medicine, University of Gezira, Wad Medani, Sudan; University of Medicine and Pharmacy at Ho Chi Minh City, Ho Chi Minh City, Vietnam; Faculty of Medicine, South Valley University, Qena, Egypt; Department of Infectious Disease, Hanoi Medical University, Hanoi, Vietnam; Traditional Medicine Hospital, Ministry of the Public Security, Hanoi, Vietnam; Resident Medical Officer, Southern Adelaide Local Health Network, SA, Australia; Rajarshee Chhatrapati Shahu Maharaj Government Medical College, Kolhapur, India; Infectious Disease Hospital, Kano, Nigeria; Central Department of Microbiology, Tribhuvan University, Kathmandu, Nepal; National Hospital of Traditional Medicine, Hanoi, Vietnam; University of Medicine and Pharmacy at Ho Chi Minh City, Ho Chi Minh City, Vietnam; Department of Infectious Disease, Hanoi Medical University, Hanoi, Vietnam

**Keywords:** perceived stress status, COVID-19, mental health, quarantine, survey

## Abstract

**Aims:** Understanding of the perceived stress and coping strategies adopted by people is important for contemplating the consequences of a pandemic on mental health of people globally. In this study, we intended to assess the perceived stress status under quarantine/isolation globally during the COVID-19 pandemic.

**Methods:** This is a multicentre, multinational cross-sectional study that recruited isolated/quarantined individuals suspected or confirmed to have COVID-19 to assess the psychological impact of the quarantine/isolation experience by answering a survey distributed online.

**Results:** The study was conducted across 63 participating countries, gaining 1,871 valid responses. There was a higher proportion of female participants in the Moderate to High Perceived Stress Scores (MH-PSS) group compared to the Low Perceived Stress Score group (66.0 vs. 52.0%) and a higher proportion of individuals whose marital status was single had MH-PSS (57.1%). Also, individual's religion (Christian, Hindu, and Muslim), no formal education level, being exposed to a confirmed or suspected COVID-19 patient, being forced to be quarantined/isolated, uncomfortable feeling during quarantine period may significantly increase the risk of MH-PSS (*p* < 0.05).

**Conclusions:** Many factors can predict stress in COVID-19 pandemic including female sex, being single, religion, no formal education, involuntary quarantine, location and reason of quarantine/isolation, and place of exposure.

## Introduction

The COVID-19 pandemic, which emerged in Wuhan, China, has rampantly spread to various countries, territories, and areas globally and has been one of the most serious pandemic over more than 100 years following the influenza pandemic in 1918 (1). Apart from the burden on the healthcare industry, the COVID-19 pandemic has significantly affected the economy worldwide (2). In response to the socio-economic burden caused by this pandemic, governments in many countries launched policies and guidelines quickly in order to ascertain control over this pandemic (2). In the absence of any definitive treatment and vaccination available against SARS-CoV-2, the causative organism; health officials and organizations worldwide have asserted the necessity of non-pharmacological interventions with imposition, if indispensable to ascertain control over the ongoing pandemic (3). Some of these non-pharmacological interventions included social/physical distancing and lockdowns with isolation of COVID-19 positive and suspected COVID-19 patients, quarantine of exposed individuals, travel limitations, closure of educational institutions and workplaces, prohibition of mass gatherings, rapid testing, proper allocation and use of personal protective equipment and maintenance of personal hygiene (3, 4).

In China, particularly in Hubei province, early measures taken by the government consisted of rigorous lockdown with travel restrictions, and social distancing measures. Other measures included rapid case detection with immediate isolation and quarantine measures for the infected and suspected cases with medical observation for all the contacts. These measures have had a positive impact on controlling the COVID-19 outbreak (4–8).

The combined non-pharmacological interventions had a substantial effect on the reduced transmission of COVID-19 across China (4). Similarly, Vietnam, which was the first nation to delineate human-to-human transmission outside China, successfully controlled the outbreak using extensive control measures without enforcing a strict lockdown (9). Vietnam instituted rapid isolation and detection of primary and secondary cases and immediate quarantine of their contacts to curb transmission. Suspected cases in Vietnam were recognized and quarantined based on their epidemiological risk of infection. In Vietnam, more than 200,000 people have been quarantined for 14 days (10). Thus, non-pharmaceutical interventions have been very effective in controlling the COVID-19 pandemic. Such non-pharmaceutical interventions have also affected the mental health of people worldwide. A recent study done on 1,784 school students in China, showed that closure of schools as a part of lockdown and social distancing guidelines affected the mental health of the students with a slightly increased prevalence of depressive and anxiety symptoms (11). Another study done on healthcare workers in Italy showed that they experienced increased symptoms of depression, post-traumatic stress symptoms, insomnia, and perceived stress (12). These findings are in congruence with the past pandemics such as Middle East Respiratory Syndrome (MERS) and Severe Acute Respiratory Syndrome (SARS) (13–15). Amongst the non-pharmaceutical interventions, quarantine and isolation have been found to be the most associated with significant psychological impact (16). Quarantine and isolation both involve separation from loved ones, lack of freedom, and boredom which can affect mental health as seen in the past pandemics (16). A study done on 170 people who were self-isolated at home during the COVID-19 outbreak in China showed that anxiety and stress of isolated individuals were at higher levels and quality of sleep was low (17). However, there is a paucity of evidence quantifying perceived stress and peoples' coping strategies adopted during confinement periods in isolation/quarantine facilities globally. Understanding of the perceived stress and coping strategies adopted by people is important for contemplating the consequences of a pandemic on mental health of people globally. The findings can help health officials in drafting policies and implementing targeted measures to reduce psychological trauma faced by quarantined/isolated people during a pandemic. In this study, we intended to assess the perceived stress status of quarantine/isolation globally during the COVID-19 pandemic and examine the various correlates involved in dealing with the perceived stress.

## Methods

### Study Design and Participants

This is a multicenter, multinational cross-sectional study that recruited isolated/quarantined individuals suspected or confirmed to have COVID-19 (from May to June 2020) to assess the psychological impact of the quarantine/isolation experience on people in quarantine/isolation areas all over the world and evaluate the ways they confronted stress during that period. Both healthcare workers and non-healthcare workers were included. The survey was distributed online in two ways; the first was distributed directly by collaborators to a convenience sample of patients in quarantine/isolation centers worldwide. The second was distributed by collaborators using a snowball sampling technique, and the collaborators acting as gatekeepers on various social media platforms promoting the survey.

The study participants were from the following countries: Afghanistan, Albania, Angola, Bangladesh, Belgium, Bolivia, Bosnia, Brazil, Canada, Chile, China, Czech Republic, Denmark, Ecuador, Egypt, El Salvador, France, Germany, Greece, Honduras, Hungary, India, Indonesia, Italy, Japan, Jordan, Kenya, Korea, Kosovo, Lebanon, Libya, Luxembourg, Malaysia, Mexico, Nepal, Netherlands, Nigeria, Oman, Pakistan, Palestine, Peru, Philippines, Poland, Portugal, Puerto Rico, Qatar, Romania, Russia, Saudi Arabia, Scotland, Seychelles, Singapore, Somalia, South Africa, Spain, Sudan, Thailand, Timor-Leste, Turkey, United Arab Emirates, United Kingdom, United States, and Vietnam (Supplementary Table 1).

### Study Questionnaire

**Figure 1:**
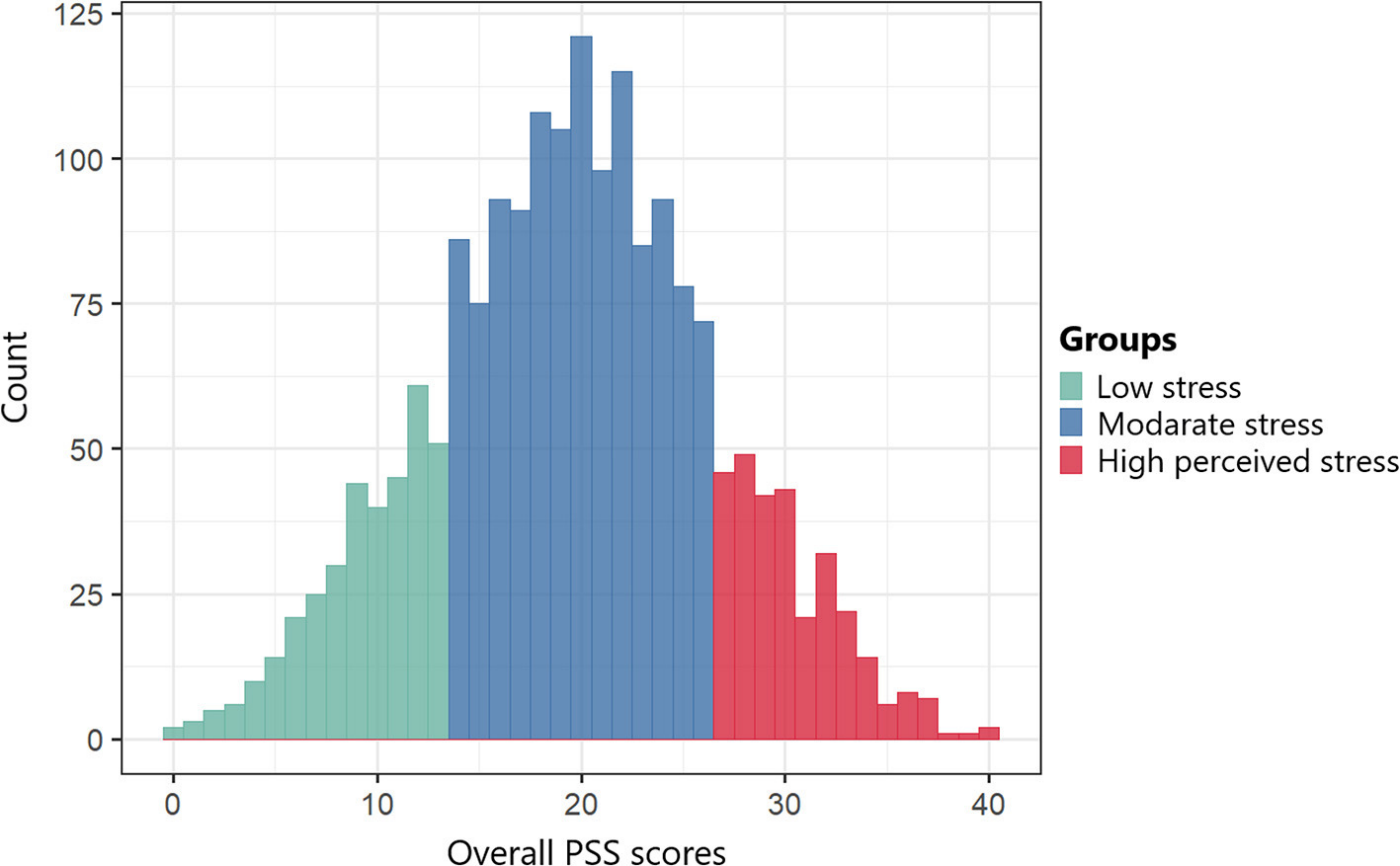
Distribution of study participants by the overall PSS scores.

The survey questionnaire included three sections. The first section was comprised of 15 questions that obtained the sociodemographic characteristics of the participants. The second section was comprised of 10 questions that obtained the quarantine/isolation information of the participants. The third section assessed the psychological impact of quarantine/isolation and coping strategies of the participants using the Perceived Stress Scale-10 (PSS-10) (18). PSS-10 is a stress assessment tool, including ten questions rated from 0 to 4. The overall PSS score can range from 0 to 40. In this study, a score from 0 to 13 was classified as low perceived stress scores (L-PSS), while a score from 14 to 40 was classified as the moderated to high perceived stress scores (MH-PSS) [Figure 1; (18)].

A panel of healthcare professionals, including two epidemiologists, one psychologist, five physicians, and five medical students, revised the questionnaire. The questionnaire was validated by a pilot survey of 30 medical students and five people who had quarantine experience during the COVID-19 outbreak. This validation ensured that all the questions were phrased clearly and appropriately for comprehension and evaluated the time needed to complete the questionnaire, and served to avoid bias that might otherwise arise.

Forward and reverse translation of the questionnaire to local languages was performed. The survey was distributed in various languages, translated by native speakers. These languages included Albanian, Arabic, Bengali, Chinese, English, Filipino, French, German, Hindi, Indonesian, Japanese, Korean, Malay, Malayalam, Nepali, Pashto, Portuguese, Russian, Spanish, Tamil, Thai, Ukrainian, Urdu, and Vietnamese. The questionnaire in each language was pretested on 3–5 native speakers and subsequently modified and validated.

Participation was voluntary, anonymous and participants had to provide informed consent on the first page of the questionnaire before accessing the rest of the questionnaire. The data from the web-based survey was extracted, kept confidential, and encrypted for analysis.

### Statistical Analysis

Descriptive statistics were performed to assess the differences in factors that influence the two categories of stress on the Perceived Stress Scores (PSS). The *T*-test was used to compare the difference in age and number of days quarantined/isolated between subjects with L-PSS and MH-PSS status, and the chi-square test was used to evaluate the remaining variables' differences. We used the logistic regression model to determine the factors related to the subject's stress. In which, the Stepwise AIC method was used to select variables related to stress status based on the optimal model. The analysis was performed using the MASS and CompareGroups packages on the R language version 3.6.0 software.

## Results

### Characteristics of Survey Participants

The study was conducted across 63 participating countries. We got a total of 6,005 responses, of which the number of valid responses was 1,871 (31.2%). The highest proportion of responses was recorded from Albania (20.6%), Vietnam (17%), and India (9%). Table 1 summarized the distribution of the survey participants' basic characteristics categorized as having either L-PSS or MH-PSS status. More than half of the participants belonged to the Asian continent (55%). Overall, the mean PSS scores of participants in the L-PSS and MH-PSS categories were 9.43 ± 2.96 and 29.6 ± 22.7, respectively. Nearly two-thirds of the participants were female (*n* = 1,174, 64%), with the mean age of 31.5 ± 10.6. Around 53.5% (*n* = 984) of participants were of Asian ethnic origin. Among the included participants, 34.4% (*n* = 637) belonged to the Muslim religion while 24.7% (*n* = 459), 19.7% (*n* = 366), 10.9% (*n* = 202), 6.9% (*n* = 128), and 3.7% (*n* = 68) belonged to Christianity, no religion, Hinduism, Buddhism, and other religions, respectively. Approximately three of every four respondents (72.5%) had their educational attainment completed up to the university level. At the time of the survey, 60% (*n* = 1,113) of the participants had employment, with only 10% (*n* = 194) having no job status at all. Among these participants, nearly one-third (*n* = 563, 30%) belonged to the healthcare profession.

**Table 1:** Basic characteristics among the survey participants across three groups of perceived stress categories.

	**Total *N* = 1,871**	**Low perceived stress score (L-PSS)**	**Moderate to high perceived stress score (MH-PSS)**	***p*-value**
		***N =* 354**	***N =* 1,507**	
**Age (year) (*****n*****=)** [Mean, SD]		32.3 (9.62)	30.7 (10.5)	0.008
**Number of quarantine days (*****n*****=)** [Mean, SD]		23.9 (19.6)	29.6 (22.7)	< 0.001
**PSS (Total points) (*****n*****=)** [Mean, SD]		9.43 (2.96)	22.0 (5.33)	< 0.001
**Gender (*****n*** **=** **1,856)**				< 0.001
Female		184 (52.0%)	990 (66.0%)	
Male		170 (48.0%)	510 (34.0%)	
**Race (*****n*** **=** **1,839)**				< 0.001
White/Caucasian		73 (21.0%)	532 (35.7%)	
Asia		243 (70.0%)	741 (49.7%)	
Hispanic/Latino		12 (3.46%)	105 (7.04%)	
Others		19 (5.48%)	114 (7.64%)	
**Religion (*****n*** **=** **1,850)**				< 0.001
No religion		112 (31.7%)	244 (16.3%)	
Buddhist		39 (11.0%)	89 (5.95%)	
Christian		58 (16.4%)	401 (26.8%)	
Hindu		32 (9.07%)	170 (11.4%)	
Muslim		101 (28.6%)	536 (35.8%)	
Others		11 (3.12%)	57 (3.81%)	
**Marital status (*****n*** **=** **1,856)**				0.004
Single		166 (47.3%)	859 (57.1%)	
Divorced/ Widowed/Separated		17 (4.84%)	57 (3.79%)	
Married/Domestic partnership		168 (47.9%)	589 (39.1%)	
**Level of education (*****n*** **=** **1,845)**				0.001
Master/PhD/Doctoral		92 (26.1%)	433 (29.0%)	
University		137 (38.8%)	676 (45.3%)	
Vocational training		34 (9.63%)	117 (7.84%)	
Primary school/Secondary school/High school		88 (24.9%)	242 (16.2%)	
No formal education		2 (0.57%)	24 (1.61%)	
**Employment status (*****n*** **=** **1,860)**				< 0.001
Full time employment		211 (59.8%)	677 (44.9%)	
Casual employment		9 (2.55%)	76 (5.04%)	
Part time employment		29 (8.22%)	111 (7.37%)	
Retired		5 (1.42%)	34 (2.26%)	
Student		69 (19.5%)	403 (26.7%)	
Unemployed		20 (5.67%)	174 (11.5%)	
Others		10 (2.83%)	32 (2.12%)	
**Average income (USD) (*****n*** **=** **1,791)**				0.052
< 250		87 (25.3%)	484 (33.4%)	
250–500		89 (25.9%)	356 (24.6%)	
500–750		56 (16.3%)	204 (14.1%)	
750–1,000		37 (10.8%)	145 (10.0%)	
Over 1,000		75 (21.8%)	258 (17.8%)	
**Main laborer in the family (*****n*** **=** **1,860)**				< 0.001
No		179 (50.6%)	949 (63.0%)	
Yes		175 (49.4%)	557 (37.0%)	
**Healthcare worker (*****n*** **=** **1,851)**				0.091
No		232 (65.7%)	1,056 (70.5%)	
Yes		121 (34.3%)	442 (29.5%)	
**Reason for quarantine/isolation (*****n*** **=** **1,773)**				< 0.001
F0		61 (17.3%)	151 (10.6%)	
F1		75 (21.3%)	205 (14.4%)	
F2/F3/F4		89 (25.3%)	423 (29.8%)	
I live, stay or work at a place nearby a confirmed COVID-19 patient		81 (23.0%)	408 (28.7%)	
I returned from affected geographic areas		46 (13.1%)	234 (16.5%)	
**Place of exposure (*****n*** **=** **1,546)**				< 0.001
In hospital		96 (31.3%)	265 (21.4%)	
At home		18 (5.86%)	98 (7.91%)	
At hotel/ At a hall, concert, cinema		2 (0.65%)	39 (3.15%)	
At workplace		34 (11.1%)	185 (14.9%)	
During travel by airplane/by bus/by taxi/by train		28 (9.12%)	159 (12.8%)	
I do not know the source of my exposure		73 (23.8%)	303 (24.5%)	
Others		56 (18.2%)	190 (15.3%)	
**Place of isolation (*****n*** **=** **1,762)**				< 0.001
At home		196 (57.8%)	1,098 (77.2%)	
At the designated place by the Government		52 (15.3%)	178 (12.5%)	
In hospital		91 (26.8%)	147 (10.3%)	
**Which of the following was true about your quarantine/isolation? (*****n*** **=** **1,756)**				< 0.001
I was forced to quarantine/isolated		38 (11.3%)	330 (23.2%)	
I was voluntarily quarantined/isolated		297 (88.7%)	1,091 (76.8%)	
**Who are you quarantined with (*****n*** **=** **1,737)**				0.007
No, only me		212 (63.3%)	779 (55.6%)	
Family		97 (29.0%)	535 (38.2%)	
Others		26 (7.76%)	88 (6.28%)	
**Comfortable in isolation time (*****n*** **=** **1,824)**				< 0.001
Not at all		13 (3.71%)	103 (6.99%)	
A little bit		35 (10.0%)	195 (13.2%)	
Moderately		116 (33.1%)	592 (40.2%)	
Quite a bit		104 (29.7%)	402 (27.3%)	
Extremely		82 (23.4%)	182 (12.3%)	
**Continent (*****n*** **=** **1,854)**				< 0.001
Africa		12 (3.39%)	81 (5.40%)	
America		21 (5.93%)	138 (9.20%)	
Asia		256 (72.3%)	785 (52.3%)	
Europe		65 (18.4%)	496 (33.1%)	

In response to the question of the place of exposure among the participants, 37.5% (*n* = 580) reported hospital or workplace as their source of exposure, and 24.3% (*n* = 376) could not identify the source, while 12.1% (*n* = 187) individuals got exposure while traveling. Nearly three-fourths of the individuals (73%, *n* = 1,294) kept themselves isolated at home either alone (56.4%, *n* = 991) or with family (36.0%, *n* = 632). The majority of the participants (79%, *n* = 1,388) remained in quarantine/isolation voluntarily, and 81% (*n* = 1,478) of participants reported their comfort to be ranging from extreme to quite a6 bit while being in isolation.

### Factors Associated Across Different Level of Perceived Stress Score Status

All the explored characteristics in Table 1, across the L-PSS and MH-PSS categories, were statistically significantly different except average income and healthcare worker characteristics. Table 1 showed that the increase in the number of quarantine days was associated with an increased level of perceived stress across L-PSS and MH-PSS categories, reporting a mean of 23.9 ± 19.6 days and 29.6 ± 22.7 days, respectively (*p* < 0.001). There was a higher proportion of female participants in the MH-PSS compared to L-PSS category (66.0 vs. 52.0%). Among the Asian ethnic respondents, a higher proportion of respondents was reported in the L-PSS group (70.0%), while the White/Caucasian respondents reported a higher proportion of respondents in the MH-PSS group (35.7%). The study estimated that a higher proportion of individuals whose marital status was single experienced high stress (57.1%). People with no formal education were more likely to have higher stress (*p* < 0.001).

Interestingly, there was a higher proportion of respondents in the MH-PSS category than the L-PSS category group among non-healthcare participants (70.5 vs. 65.7%) in contrast to the findings among healthcare participants (29.5 vs. 34.3%). The study found a higher proportion of respondents in the MH-PSS group than the L-PSS group among participants who underwent quarantine/isolation due to their residence or workplace being near a confirmed COVID-19 patient, returnees from affected geographic areas, and close contacts of confirmed or suspected COVID-19 cases. A higher proportion of respondents was found among the MH-PSS group compared to the L-PSS group among respondents who were unaware of their source of exposure. Also, a higher prevalence of participants having higher PSS was found among those whose place of isolation was home compared with those residing in a hospital or at a government-designated place.

### Predictors of MH-PSS Among the Survey Participants

Table 2 illustrated the explanatory variables in the multivariate ordinal regression model that contributed to predicting MH-PSS among the included survey participants. In the regression result, male respondents were more likely to have MH-PSS (OR: 1.43, 95% CI: 1.02–2.02, *p* = 0.038) compared to their female counterparts. The study found that an individual's religion may increase the odds of MH-PSS compared to the participants who identified themselves as having no religion. Compared to those with no religious affiliation, the odds of having MH-PSS were significantly higher among those who identified as Hindus (OR: 4.40, 95% CI: 2.41–78.32), followed by Christians (OR: 4.11, 95% CI: 2.46–7.03), and Muslims (OR: 2.47, 95% CI: 1.62–3.80) (*p* < 0.001). Individuals who possessed no formal education were more likely to have MH-PSS (OR: 9.60, 95% CI: 1.74–180.51, *p* = 0.035) compared to individuals who had attained higher educational status. Students (OR: 1.63, 95% CI: 1.07–2.51, *p* = 0.023) and those on casual employment (OR: 2.56, 95% CI: 1.08–7.20, *p* = 0.047) had a significantly higher odds of having MH-PSS when compared to full-time employed respondents. The index case (F1) had about 1.96 times higher odds to be in the MH-PSS category (OR: 1.96, 95% CI: 1.17–3.28, *p* = 0.011) than the corresponding F0 case. The study also found that participants had 1.99 times increased odds of MH-PSS when the workplace (OR: 1.99, 95% CI: 1.13–3.61, *p* = 0.02) was designated as the place of exposure to COVID-19 compared to been exposed in the hospital setting. Participants who were voluntarily isolated/quarantine (OR: 0.51, 95% CI: 0.31–0.80, *p* = 0.005) were less likely to have MH-PSS category than participants who were forced to quarantine/isolation. There were decreased odds of MH-PSS category when an individual reported that they remained comfortable during the period of isolation/quarantine. Participants reporting that they were not at all comfortable during isolation/quarantine time had the highest likelihood of higher stress (OR: 3.56, 95% CI: 1.44–10.20, *p* = 0.010) compared to those that were extremely comfortable during isolation.

**Table 2:** Multivariable logistic regression analysis for factors associated with perceived stress among the quarantined/isolated participants.

**Variables**	**Univariate analysis**		**Multivariable analysis**	
	**OR (95% CI)**	***p*-value**	**OR (95% CI)**	***p*-value**
**Age (10 years)**	1.17 (1.06–1.31)	0.003	1.20 (1.06–1.38)	0.007
**Gender**				
Male	*Reference*	*Reference*		
Female	1.68 (1.27–2.24)	< 0.001	1.43 (1.02–2.02)	0.038
**Religion**				
No religion	*Reference*	*Reference*		
Buddhist	1.63 (0.98–2.77)	0.067	1.38 (0.77–2.52)	0.280
Christian	4.84 (3.06–7.84)	< 0.001	4.11 (2.46–7.03)	< 0.001
Hindu	2.87 (1.69–5.04)	< 0.001	4.40 (2.41–8.32)	< 0.001
Muslim	2.53 (1.76–3.65)	< 0.001	2.47 (1.62–3.80)	< 0.001
Others	2.93 (1.32–7.33)	0.013	2.44 (1.04–6.48)	0.053
**Level of education**				
Master/ PhD/Doctoral	*Reference*	*Reference*		
University	1.03 (0.72–1.47)	0.851	1.28 (0.86–1.90)	0.224
Vocational training	0.73 (0.42–1.28)	0.261	1.16 (0.62–2.20)	0.654
Primary school/Secondary school/High school	0.57 (0.38–0.87)	0.009	0.65 (0.39–1.09)	0.101
No formal education	4.55 (0.92–82.32)	0.143	9.60 (1.74–180.51)	0.035
**Employment status**				
Full time employment	*Reference*	*Reference*		
Casual employment	2.59 (1.18–6.83)	0.031	2.57 (1.08–7.20)	0.047
Part time employment	0.95 (0.59–1.59)	0.843	0.68 (0.39–1.21)	0.176
Retired	1.95 (0.66–8.37)	0.286	3.51 (1.02–16.73)	0.070
Student	1.57 (1.10–2.27)	0.015	1.63 (1.07–2.51)	0.023
Unemployed	2.89 (1.50–6.31)	0.003	2.13 (1.01–5.00)	0.061
Others	0.88 (0.33–2.74)	0.804	0.80 (0.26–2.83)	0.717
**Reason for quarantine/isolation**				
I was F0	*Reference*	*Reference*		
I was F1	1.66 (1.06–2.58)	0.026	1.96 (1.17–3.28)	0.011
I was F2/F3/F4	2.15 (1.43–3.23)	< 0.001	2.05 (1.27–3.29)	0.003
I live, stay or work at a place nearby a confirmed COVID-19 patient	2.78 (1.72–4.58)	< 0.001	1.33 (0.73–2.45)	0.351
I returned from affected geographic areas	2.72 (1.65–4.58)	< 0.001	2.07 (1.15–3.80)	0.017
**Place of exposure**				
In hospital	*Reference*	*Reference*		
At home	1.07 (0.60–1.99)	0.826	1.58 (0.81–3.20)	0.194
At hotel/At a hall, concert, cinema	8.71 (1.83–156.35)	0.035	12.36 (2.39–227.72)	0.017
At workplace	1.63 (1.00–2.75)	0.057	1.99 (1.13–3.61)	0.020
During travel by airplane/by bus/by taxi/by train	1.40 (0.85–2.38)	0.200	1.95 (1.10–3.53)	0.024
I do not know the source of my exposure	0.74 (0.51–1.08)	0.120	1.23 (0.77–1.96)	0.383
Others	0.89 (0.55–1.46)	0.643	0.94 (0.54–1.67)	0.842
**Which of the following was true about your quarantine/isolation?**				
I was forced to quarantine/isolated	*Reference*	*Reference*		
I was voluntarily quarantined/isolated	0.44 (0.28–0.66)	< 0.001	0.51 (0.31–0.80)	0.005
**Comfortable in isolation time**				
Extremely	*Reference*	*Reference*		
Quite a bit	1.51 (1.00–2.27)	0.050	1.40 (0.88–2.24)	0.158
Moderately	1.88 (1.26–2.81)	0.002	2.09 (1.32–3.30)	0.001
A little bit	1.91 (1.12–3.35)	0.020	2.31 (1.24–4.41)	0.010
Not at all	4.15 (1.82–11.26)	0.002	3.56 (1.44–10.20)	0.010
**Continent**				
Asia	*Reference*	*Reference*		
Africa	2.90 (1.33–7.62)	0.015	2.50 (1.05–7.01)	0.055
America	1.90 (1.16–3.26)	0.015	1.60 (0.88–3.03)	0.133
Europe	2.96 (1.97–4.61)	< 0.001	2.58 (1.51–4.52)	0.001

## Discussion

We examined the various factors attributed to stress among individuals who were quarantined or isolated during the COVID-19 pandemic through our survey. There appeared to be a strong correlation between the number of days spent in quarantine and the PSS score, with a calculated risk of 0.7% increase in the PSS score with each additional day of quarantine. The impact of the duration of quarantine on mental health has been studied previously, particularly during the SARS outbreak, which demonstrated that prolonged quarantine periods were associated with symptoms of post-traumatic stress, anger, avoidance behavior, and overall poor mental health (19–21). In their study during the early days of the COVID-19 pandemic, Lu et al. (22) observed a similar relationship between quarantine duration and anxiety/depression by monitoring the activity of quarantined individuals on their Twitter accounts. The authors collectively analyzed 214,874 tweets from 1,278 quarantined individuals and 250,198 tweets from 1,278 individuals who served as controls. Besides, they also discovered fluctuations in the psychological state of the individuals, which was characterized by an increase in anxiety/depression at the start of the quarantine, followed by a gradual decrease, and a resurgence in anxiety/depression beyond 14 days of quarantine (22). Hawryluck et al. (19) demonstrated a significant rise in post-traumatic stress symptoms in individuals quarantined for more than 10 days during the SARS outbreak as opposed to individuals quarantined <10 days (19). In perspective, the number of days spent in quarantine was much higher among our study participants in comparison to previous studies, with the average duration of quarantine being 28.5 days, most likely attributed to the long incubation period associated with the illness (23). Regardless, the correlation between quarantine duration and impact on mental stress was demonstrated despite this aspect.

Differences in gender distribution were also observed across the groups of varying PSS scores. In general, females were more likely to report being stressed during the quarantine than males. This is in line with previous studies, which have also demonstrated a gender gap in the mental health impact caused by quarantine during outbreaks. Song et al. discovered a similar trend in their study among Chinese individuals in quarantine during the COVID-19 pandemic. In addition, the authors identified that the risk factors for stress were different for males and females. Among males, occupations with unstable income, higher education status, and quarantine duration were the most common risk factors.

In contrast, the need for information about the pandemic, worsening of the outbreak locally, and health problems during the quarantine were the key risk factors among females. Interestingly, the authors noted that males were less adaptive and had a lower tendency to recognize the need for psychological support when compared with females; however, they were more likely to seek medical care in response to infective symptoms (24). The observed gender differences are postulated to be due to the variation in psychological, physiological, and cognitive processes between the two sexes and their consequent response to stressful situations (25–27).

We also observed a relationship between relationship status and PSS scores, with single individuals being at the most significant risk of higher stress. This is in line with the findings from a recent study by Pieh et al. (28) during the COVID19 pandemic, where the authors demonstrated that participants with a good quality relationship scored significantly better on all mental health scales than individuals who were not in a relationship of any kind or those who were in a poor-quality relationship, in that order. The authors concluded that while being in a relationship in itself did not determine mental health status during quarantine, the quality of the relationship certainly played a role (28).

Contrary to expectations, MH-PSS was observed more frequently among individuals with religious beliefs compared to those with no religious beliefs. However, it is essential to note that nearly 80% of our study population identified with a religion, which may have contributed to this unexpected result. Yildirim et al. (29) found that positive religious coping mediated a greater meaning in life, which reduced loneliness during the coronavirus crises in Turkey. In line with this, the Thomas and Barbato (30) published similar findings demonstrating that positive religious coping was inversely related to depressive symptoms and a history of psychological disorders during the COVID-19 pandemic. Interestingly, the authors also found that Muslims had higher levels of positive religious coping than the Christians in their sample population. In our study, we did not assess the extent of religious involvement or the various religious coping tools/ strategies used by participants, which is believed to have played a role in our findings. In our study, we found that a higher educational status was associated with increased stress. The data on the link between educational status and psychological stress during quarantine is obscure. In a study among horse owners during an equine influenza outbreak, Taylor et al. observed that individuals with lower formal educational qualifications were at an increased risk of negative psychological impacts (31). On the other hand, Hawryluck et al. reported that the level of education did not affect both the understanding of quarantine requirements or the psychological impact of quarantining (19). In contrast, Song et al. noted that a higher educational status correlated positively with stress levels during the COVID-19 pandemic (24). Regardless, the need for information about the illness from public health officials and its clarity have been reported to influence the mental health of individuals in quarantine/isolation (32). The educational status of the individual may perhaps play a role in the understanding of this information and, therefore, affect the psychosocial impact of quarantine.

The observed relationship between being a confirmed case of infection, or the index case, in a family and the high levels of perceived stress, has been documented in previous studies. In a study by Jeong et al. during the MERS outbreak, it was reported that individuals who were isolated due to suspicion or confirmation of infection were more likely to manifest anxiety if they experienced symptoms characteristic of the infection and link it to the infection even if the suspected exposure was several months prior to the presentation of these symptoms (33). Studies also show that individuals who have been exposed to the risk of infection tend to worry about spreading the infection to their family members and others who have come into contact with them (32–34).

Recent studies in this area have identified other pre-existing individual characteristics that contribute to stress during pandemics. Osimo et al. (35) investigated the influence of personality traits on stress response during COVID-19 lockdowns and reported that individuals with a lower resilience and emotional stability and higher alexithymia had a poorer emotional response during home containment. Similarly, Moccia et al. (36), through their study on the Italian population during the COVID-19 pandemic, found that individuals with cyclothymic, depressive, and anxious temperaments and those who had a “Need for approval” style as per the Attachment Style Questionnaire (ASQ) were at higher risk of moderate-to-severe psychological distress; whereas ASQ “Confidence” and ASQ “Discomfort with closeness” was found to be protective traits. Another study conducted by Fiorillo et al. (37) during March to May 2020 in Italian population showed individuals with known mental disorders and physical disease had worse levels of depression and anxiety symptoms while individuals with cohabiting people, living with more number of family members and having higher level of satisfaction with one's own life were found to be protective against development of psychiatric symptoms.

Based on our search of the literature, the strength of our study is its multinational population, with participants from a total of 63 countries, making it one of the most diverse of recent publications on the relationship between quarantine during the COVID-19 pandemic and its psychological impact. We identified some of the key risk factors that contribute to anxiety/stress during the period of quarantine/isolation through our analysis. As with any study, there are several limitations to our research. While our survey reached participants of multiple countries, providing a heterogeneous multinational sample, the overall number of responses was relatively low, limiting the size of the study. In addition, we aimed to keep the survey at a length that was palatable to respondents. We were unable to assess several other factors such as pre-existing mental health illnesses, religious coping, individual temperament and self-control, ability to work from home, and living conditions. We believe that our study captures some of the major risk factors for mental stress during quarantine/isolation, specific to the COVID-19 pandemic, and paves the way for further research in this area. The findings of this study should be considered when dealing with psychosocial issues that arise during infectious outbreaks and help identify high-risk groups for negative psychological impact for early intervention.

Taken together our findings, many factors can predict stress in pandemics such as COVID-19, including female sex, being single, higher education status, and being of the non-healthcare profession. These and similar factors should be considered in handling stress and in managing future pandemics.

## Data Availability Statement

The raw data supporting the conclusions of this article will be made available by the authors, without undue reservation.

## Ethics Statement

The studies involving human participants were reviewed and approved by ethical approval was obtained from the ethical committee of the IRB of Nagasaki University (Ref. No. 117; Approval number: NU_TMGH_2020_117_1). The patients/participants provided their written informed consent to participate in this study.

## Consent to Publish

All authors give the full consent to publish this study.

## Patient and Public Involvement

We collected data from the individuals who were quarantined/isolated after being suspected or confirmed to have contracted COVID-19. However, they have not involved directly in the setting of the research question or outcome measures. They did not have any role in designing or implementing this work or interpretation of the results.

## Author Contributions

NH, ND, and LM: design of the idea and study workflow. ND, SM, TG, VT, LL, and PT: data acquisition. ND, SM, LM, AH, TG, LT, AR, FD, VT, and PT: data interpretation. LM, LT, and VT: final analysis. SM, AH, AR, and SD: drafting of the manuscript. NH, ND, LM, and SD: critical revision. All authors contributed to the final approved the final version.

## Conflict of Interest

The authors declare that the research was conducted in the absence of any commercial or financial relationships that could be construed as a potential conflict of interest.
