# Supplementary material for: Perceived Stress of Quarantine and Isolation During COVID-19 Pandemic: A Global Survey
Source: Front Psychiatry. 2021 May 25;12:656664. doi: 10.3389/fpsyt.2021.656664 (PMC8186534; doi:10.3389/fpsyt.2021.656664)
Supplement: Supplementary file 1 [file Data_Sheet_1.docx]

**AUTHORS:**

Nguyen Tien Huy^*^ (School of Tropical Medicine and Global Health, Nagasaki University, Nagasaki, Japan, tienhuy@nagasaki-u.ac.jp), Nguyen Tran Minh Duc^*^ (University of Medicine and Pharmacy at Ho Chi Minh City, Ho Chi Minh City, Vietnam, minhduc1298@gmail.com), Shamael Thabit Mohammed Alhady^*^ (Faculty of Medicine, University of Gezira, Wad Medani, Sudan, shamaelthabit4321@gmail.com), Luu Ngoc Mai^*^ (University of Medicine and Pharmacy at Ho Chi Minh City, Ho Chi Minh City, Vietnam, ngmai3288@gmail.com), Amr K. Hassan (Faculty of Medicine, South Valley University, Qena, Egypt, dramrhassan@yahoo.com), Tran Van Giang (Department of Infectious Disease, Hanoi Medical University, Hanoi, Vietnam, giangminh08@gmail.com), Le Van Truong (Traditional Medicine Hospital, Ministry of the Public Security, Hanoi, Vietnam, bs.vantruong@gmail.com), Rohanti Ravikulan (Resident Medical Officer, Southern Adelaide Local Health Network, SA, Australia, r.rohanti@gmail.com), Akshay Raut (Rajarshee Chhatrapati Shahu Maharaj Government Medical College, Kolhapur, India, akshayraut2610@gmail.com), Farouq Muhammad Dayyab (Infectious Disease Hospital, Kano, Nigeria, farouqmuhd@yahoo.com), Shyam Prakash Durme (Central Department of Microbiology, Tribhuvan University, Kathmandu, Nepal, sp.durme@gmail.com), Vu Thi Thu Trang (National Hospital of Traditional Medicine, Hanoi, Vietnam, dr.vutrang@gmail.com),Le Quang Loc (University of Medicine and Pharmacy at Ho Chi Minh City, Ho Chi Minh City, Vietnam, lequangloc.md@gmail.com), Pham Ngoc Thach (Department of Infectious Disease, Hanoi Medical University, Hanoi, Vietnam, phamngocthachnhtd@gmail.com)

**E-mail and ORCID:**

**NTH: tienhuy@nagasaki-u.ac.jp; 0000-0002-9543-9440**

**NTMD: minhduc1298@gmail.com; 0000-0002-9333-7539**

**STMA: shamaelthabit4321@gmail.com; 0000-0001-7463-3300**

**LMN: ngmai3288@gmail.com; 0000-0002-8129-1764**

**AKH : dramrhassan@yahoo.com ; 0000-0002-8281-9268**

**TVG: giangminh08@gmail.com; 0000-0002-3781-2331**

**LVT: bs.vantruong@gmail.com; 0000-0002-7474-0453**

**RR: r.rohanti@gmail.com; 0000-0002-6402-1279**

**AR: akshayraut2610@gmail.com; 0000-0002-0210-989**

**FMD: farouqmuhd@yahoo.com; 0000-0001-8920-6483**

**SPD: sp.durme@gmail.com; 0000-0002-4072-0745**

**VTTT: dr.vutrang@gmail.com; 0000-0001-7152-4299**

**LQL: lequangloc.md@gmail.com; 0000-0001-6363-9862**

**PNT: phamngocthachnhtd@gmail.com**

***Nguyen Tien Huy, Nguyen Tran Minh Duc, Shamael Thabit Mohammed Alhady, and Luu Ngoc Mai contributed equally to this article.**

**Author for correspondence: Nguyen Tien Huy, E-mail: tienhuy@nagasaki-u.ac.jp**

**COLLABORATORS:**

Shyam Prakash Durme (Central Department of microbiology, Tribhuvan University, Kathmandu, Nepal; Institute of Tropical Medicine, Nagasaki University, Japan)(1a), Kamal Ranabhat(1b)(Ministry of Health and Population, Kathmandu, Nepal; Institute of Medicine, Tribhuvan University, Kathmandu, Nepal), Irida Dajti(1c)(UHOG 'Koço Gliozheni'), Ian Christopher Naungayan Rocha(1d) (School of Medicine, Centro Escolar University, Manila, Philippines), Venkatesh U(1e)Department of Community Medicine, Vardhman Mahavir Medical College & Safdarjung Hospital, New Delhi), Md Ariful Haque(1f)(Department of Orthopedic Surgery, Yan an Hospital Affiliated to Kunming Medical University, Kunming, Yunnan, China), Graca Jaqueline Vanessa Morena(1g)(Faculty of Medicine, Trisakti University - Jakarta, Indonesia & America Evangelical University, Los Angeles CA), Ton That Khanh(1h)(Faculty of Medicine, School of Medicine and Pharmacy, Da Nang University, Da Nang City, Vietnam), Filipa Lucas(1i)(Faculdade de Medicina da Universidade de Lisboa), Ahmed Hisham Mohamed Hamed(1j)(Faculty of Medicine, Cairo University, Cairo, Egypt), Irfan Ullah(1k)(Kabir Medical College, Gandhara University, Peshawar, Pakistan), Dmytro Pavlenko(1l)(Department of Ophthalmology, Bogomolets National Medical University, Kyiv, Ukraine), Sze Jia Ng(1m)(School of Medical Sciences, Universiti Sains Malaysia, Kelantan, Malaysia), Andrés Sebastián Estrella Lopez(1n)(Faculty of Medicine, Pontificia Universidad Católica del Ecuador)

Kevin Thurston Crispino(2a)(Health Emergency Management Bureau, Department of Health, Manila, Philippines), Enxhi Vrapi(2b)(UHOG 'Koço Gliozheni'), Jola Kërpaçi(2c)(UHOG 'Koço Gliozheni'), Brianda del Pilar Gómez Olvera(2d)(Autonomous University of Mexico State), Adriana Viola Miranda(2e)(Faculty of Medicine, University of Indonesia, Jakarta, Indonesia), Rifath Jahan Antora(2f) (Qilu Hospital, Shandong University, Jinan, Shandong, China), Roman Pavlenko(2g)(Bogomolets National Medical University, Kyiv, Ukraine), Rachel Silency Aritonang(2h)(Faculty of Medicine, Trisakti University - Jakarta, Indonesia & Yadika General Hospital, Indonesia), Iryna Kudlatska-Tyshko(2i)(Shupyk National Medical Academy of Postgraduate Education, Kyiv, Ukraine), Suhir Alsuwiyah(2j)(Faculty of Medicine, University of Tripoli, Libya), Zair Hassan(2k)(Cardiology dpt, Lady Reading Hospital), Iftikhar Ali(2l)(Paraplegic Center, Hayatabad Peshawar), Nahida AL HABAJ(2m)(Faculty of medicine, University of Tours, France), Mariia Pavlenko(2n)(Kyiv City Pediatric Diagnostic Center, Kyiv, Ukraine)

Juwie Chuah(3a)(School of Medical Sciences, Universiti Sains Malaysia, Kelantan, Malaysia), Yi Liang Lim(3b)(School of Medical Sciences, Universiti Sains Malaysia, Kelantan, Malaysia), Yap Siang Jee(3c)(School of Medical Sciences, Universiti Sains Malaysia, Kelantan, Malaysia), Li Chuin Chong(3d)(School of Data Sciences, Perdana University), Samah Melhassan(3e)(Alneelain University, Faculty of Medicine)

Asad Ali Khan (Department of Internal Medicine, Khyber Teaching Hospital, Peshawar, Pakistan)*, Ahmad Taysir Atieh Qarawi*(Lower Westchester Medical Associates, P.C., Mount Vernon, NY 10550, USA), Rejwana Haque Pial*(National Institute of Preventive and Social Medicine), Salma Elnoamany*(Faculty of Medicine, Menoufia University, Menoufia, Egypt ), Mawada fath Alrahman*(University of Bakhat alruda, Sudan), Ahmed Abdelfattah El Gharably*(Faculty of Medicine, South Valley University, Qena, Egypt), Jeza Muhamad Abdul Aziz*(Medical Laboratory Science, College of Health Sciences, University of Human Development, Sulaymaniyah, Kurdistan region-Iraq. Baxshin Research Center, Baxshin Hospital, Sulaymaniyah, Kurdistan Region-Iraq), Kirellos Said Abbas*(Faculty of Medicine, Alexandria University, Alexandria, Egypt.), Fatma A.Monib*(Faculty of Medicine, Assiut University, Assiut, Egypt.), Aliaa Effat Said* (Faculty of Medicine, Assiut University, Assiut, Egypt.), Shehab Fathy Ahmed*(Faculty of Medicine, Assiut University, Assiut, Egypt), Ziad Hassan Hamed*(Faculty of Medicine, Assiut University, Assiut, Egypt.), Mariam Albatoul Nageh* (Faculty of Medicine, Assiut University, Assiut Egypt), Esraa Sayed*(Faculty of Medicine, Assiut University, Assiut, Egypt.), Mosa Shibani*(Faculty of Medicine, Syrian Private University. Damascus, Syrian Arab Republic.), Hlma Ismail*(Faculty of Medicine, Syrian Private University. Damascus, Syrian Arab Republic.), Mhd Amin Alzabibi*(Faculty of Medicine, Syrian Private University. Damascus, Syrian Arab Republic.), Bisher Sawaf*(Internal Medicine Department, Hamad General Hospital, Hamad Medical Corporation, Doha, Qatar), Hoda Aly Mohamed Omran*(Faculty of Medicine, Alexandria University, Alexandria, Egypt.), Chiristine Samuel Rezq*(Faculty of Medicine, Alexandria University, Alexandria, Egypt.), mohamed ibrahim abdo ibrahim*(Faculty of Medicine, Alexandria University, Alexandria, Egypt.), Marina Samy Ragheb*(Faculty of Medicine, Alexandria University, Alexandria, Egypt)*, Boughalem Younes* (Faculty of Medicine and Pharmacy, Cadi Ayyad University, Marrakesh, Morocco), Rachel Silency Aritonang*((Faculty of Medicine, Trisakti University - Jakarta, Indonesia & Yadika General Hospital, Indonesia), Atsuko Imoto*(School of Tropical Medicine and Global Health, Nagasaki University, Japan), Kazumi Kubota*(Department of Biostatistics, Yokohama City University, Japan), Koji Aoki*(Graduate School of Nursing, Chiba University, Japan), Hemin Fatih Hama Kareem*(Department of Psychiatry, Mental Health Center, Baxshin Hospital Sulaymaniyah, Kurdistan region,Iraq), Rangin Muhamad Hussein*(Baxshin Research Center, Baxshin Hospital, Sulaymaniyah-Kurdistan region-Iraq), Mohammed Ibrahim Mohialdeen Gubari*(Department of Family and Community Medicine, College of Medicine, University of Sulaimaniyah, Sulaimaniyah, Kurdistan Region, Iraq.), Pradip Gyanwali*(Nepal Health Research Council, Kathmandu, Nepal), Meghnath Dhimal*(Nepal Health Research Council, Kathmandu, Nepal), Samir Kumar Adhikari* (Chief of National Health Emergency Operation Center, Ministry of Health and Population, Kathmandu, Nepal), Kriti Adhikari*(Nepal Health Research Council, Kathmandu, Nepal), Pallavi Koirala*(Nepal Health Research Council, Kathmandu, Nepal), Renu Bhandari Dumre*(School of Tropical Medicine and Global Health, Nagasaki University, Nagasaki, Japan), Joyce Nicole Pineda Ordóñez*(Faculty of Medicine, Catholic University of Honduras), José Tomás Ordóñez Aburto*(Faculty of Medicine, Universidad de La Frontera, Chile), Nut Koonrungsesomboon* (Department of Pharmacology, Faculty of Medicine, Chiang Mai University, Chiang Mai, Thailand), Chidchanok Ruengorn*(Department of Pharmaceutical Care, Faculty of Pharmacy, Chiang Mai University, Chiang Mai, Thailand), Surapon Nochaiwong*(Department of Pharmaceutical Care, Faculty of Pharmacy, Chiang Mai University, Chiang Mai, Thailand), Penkarn Kanjanarat* (Department of Pharmaceutical Care, Faculty of Pharmacy, Chiang Mai University, Chiang Mai, Thailand), Mingkwan Na Takuathung* (Department of Pharmacology, Faculty of Medicine, Chiang Mai University, Chiang Mai, Thailand), Somia Iqtadar* (King Edward Medical University Pakistan), Usman Ghani* (Directorate General Health Services Punjab), Sami Ullah Mumtaz* (North Medical Ward,KEMU/Mayo Hospital Lahore), Ali Dzhemiliev* (Shupyk National Medical Academy of Postgraduate Education, Kyiv, Ukraine)

**Collaborators' roles:**

 Members were divided into four groups

(1): who was a leader and translated and distributed the questionnaire

(2): who translated and distributed, or distributed only but got >60 responses

(3): who didn't translate but got more than 50 responses, And Who didn't distribute and translated more than one language

(4): who distributed only and got <50 responses

And who translated only and less than two languages

(*): means collaborators shared roles equally

**Email and ORCID**

SPD [sp.durme@gmail.com](mailto:sp.durme@gmail.com); 0000-0002-4072-0745

KR kamal_raj7@iom.edu.np; 0000-0002-3396-7440

ID irida.dajti@gmail.com; 0000-0002-8449-6829

ICNR giannisrocha@gmail.com; 0000-0002-8775-6876

VU venkatesh2007mbbs@gmail.com ; 0000-0001-8059-9364

DMAH arifulhaque58@gmail.com ; 0000-0003-4632-5153

GJVM moresoares11@gmail.com; 0000-0002-3531-0370

TTK khanhtonthat08@gmail.com; 0000-0003-3462-4244

FL Filipa-lucas@campus.ul.pt; 0000-0001-7651-7194

AHMH ahmed.hamed@students.kasralainy.edu.eg; 0000-0002-0843-532X

IU irfanullahecp2@gmail.com;[0000-0003-1100-101X](https://orcid.org/0000-0003-1100-101X)

DP pavlenkomd@gmail.com; 0000-0001-8761-9559

DSJN elaine-1_ng@outlook.com; 0000-0001-5353-6499

ASEL andresestrella823@gmail.com; 0000-0003-1929-7548

KTC kevincrispino16@gmail.com; 0000-0002-6505-2982

EV enxhivrapi@gmail.com; 0000-0002-2767-5599

JK jolamedicine16@gmail.com; 0000-0002-6893-477X

BPGO gom-olv@live.com.mx; 0000-0002-5905-8616

AVM adrianaviola99@gmail.com; 0000-0001-8548-1592

RJA [dr.rifathjahan060@gmail.com](mailto:dr.rifathjahan060@gmail.com); 0000-0003-0848-9456

RP tjroma1@gmail.com; 0000-0001-8070-5649

RSA aritonangrachel@gmail.com; 0000-0003-2702-2691

IKT irynakudlatska@gmail.com; [0000-0002-2700-1455](https://orcid.org/0000-0002-2700-1455)

SA [suhir.alhadi@gmail.com](mailto:suhir.alhadi@gmail.com); 0000-0003-1059-4431

ZH [zair.hassan7272@gmail.com](mailto:zair.hassan7272@gmail.com); 0000-0001-9789-0270

IA [Iftikharalijan@gmail.com](mailto:Iftikharalijan@gmail.com); 0000-0002-0809-7586

NAH [nahidaalhabaj@hotmail.com](mailto:nahidaalhabaj@hotmail.com); 0000-0002-2275-831X

AAK doctorasadalikhan@gmail.com ; 0000-0001-8503-3036

ATAQ [ahmadtqarawi@gmail.com](mailto:ahmadtqarawi@gmail.com); 0000-0001-7278-9889

RHP [rejwana.hp25@gmail.com](mailto:rejwana.hp25@gmail.com); 0000-0002-7012-7889

SE [Salmaalnomany6@gmail.com](mailto:Salmaalnomany6@gmail.com); 0000-0002-6415-9948

MAF mawadafath74@gmail.com ; 0000-0003-4721-8536

AAEG [ahmedmhameddd1996@gmail.com](mailto:ahmedmhameddd1996@gmail.com) ; 0000-0002-3212-5436

JMA [jeza1981@gmail.com](mailto:jeza1981@gmail.com); 0000-0002-1522-1880

KSA kirellossaid98@gmail.com; 0000-0003-0339-9339

FAEMMMM fatma.ahmed931977@gmail.com; 0000-0001-8456-1603

AES aliaaeffat30@gmail.com; 0000-0002-3828-6038

SFA shehabfathy134@gmail.com; 0000-0002-6964-247X

ZHH [zetairon@gmail.com](mailto:zetairon@gmail.com); 0000-0001-6587-8517

MAN mariam.16266094@med.aun.edu.eg; 0000-0002-7937-4229

EGA esraajamaleldeeb@gmail.com; 0000-0003-0290-5228

MS moosa.shibani@gmail.com; 0000-0002-4424-2728

HI Helmaismail997@gmail.com; 0000-0003-4329-9519

MAA ameenzabibi@gmail.com; 0000-0003-4050-3051

BS bishersawaf.94@gmail.com; 0000-0001-6751-619X

HAMO hodaomran100@outlook.com; 0000-0003-1840-3599

CSR chiristinesamuel@gmail.com; 0000-0002-7040-4268

miai mohamedibrihem2012@gmail.com; 0000-0002-5216-7545

MSR marinasamy25@gmail.com; 0000-0002-7691-2562

BY nsbm2000@gmail.com; 0000-0001-6903-5146

RSA aritonangrachel@gmail.com; 0000-0003-2702-2691

AI imoto@nagasaki-u.ac.jp; 0000-0003-2744-6160

DKK kubotaa@yokohama-cu.ac.jp

KA [koji-aoki@chiba-u.jp](mailto:koji-aoki@chiba-u.jp)

HFHK [dr.hemin@gmail.com](mailto:dr.hemn@gmail.com); 0000-0002-8119-5246

RMH [rangen991@gmail.com](mailto:rangen991@gmail.com); 0000-0001-5938-2224

MIMG [mohammed.jubari@gmail.com](mailto:mohammed.jubar@gmail.com); 0000-0002-2873-3981

PG [prgyawali@nhrc.gov.np](mailto:prgyawali@nhrc.gov.np)

KA [adhkriti@gmail.com](mailto:adhkriti@gmail.com)

PK [pallavik23@gmail.com](mailto:pallavik23@gmail.com)

MD meghdhimal2@gmail.com; 0000-0001-7176-7821

SKA [adhikarispk@gmail.com](mailto:adhikarispk@gmail.com) 0000-0002-3226-4428

RBD renu.bdumre@gmail.com; 0000-0001-8039-0309

JNPO jn.pineda94@gmail.com; 0000-0002-0953-8742

JTOA jtordonezaburto@gmail.com; 0000-0001-8705-4041

CR chidchanok.r@elearning.cmu.ac.th; 0000-0001-7927-1425

SN surapon.nochaiwong@gmail.com; 0000-0003-1100-7171

PK penkarnk@hotmail.com; 0000-0002-8160-5444

MNT k_mingkwan@hotmail.com; 0000-0003-4240-5367

SI Somia.iqtadar@gmail.com; 0000-0002-8482-9355

UG usman293eb@gmail.com

SUM [drsumumtaz@gmail.com](mailto:drsumumtaz@gmail.com)

MP kukushkinamd@gmail.com; 0000-0002-9564-6730

AD alijemilev@gmail.com

**TRANSLATION TEAMS**

**Albanian**

Irida Dajti (UHOG 'Koço Gliozheni'), Enxhi Vrapi (UHOG 'Koço Gliozheni'), Jola Kërpaçi (UHOG 'Koço Gliozheni')

**Arabic**

Kirellos Said Abbas (Faculty of Medicine, Alexandria University, Alexandria, Egypt), Fatma A. Monib (Faculty of Medicine, Assiut University, Assiut, Egypt), Aliaa Effat Said (Faculty of Medicine, Assiut University, Assiut, Egypt), Shehab Fathy Ahmed (Faculty of Medicine, Assiut University, Assiut, Egypt), Ziad Hassan Hamed (Faculty of Medicine, Assiut University, Assiut, Egypt), Mariam Albatoul Nageh (Faculty of Medicine, Assiut University, Assiut, Egypt), Esraa Sayed (Faculty of Medicine, Assiut University, Assiut, Egypt), Mosa Shibani (Faculty of Medicine, Syrian Private University. Damascus, Syrian Arab Republic), Hlma Ismail (Faculty of Medicine, Syrian Private University. Damascus, Syrian Arab Republic), Mhd Amin Alzabibi (Faculty of Medicine, Syrian Private University, Damascus, Syrian Arab Republic), Bisher Sawaf (Faculty of Medicine, Syrian Private University, Damascus, Syrian Arab Republic), Hoda Aly Mohamed Omran (Faculty of Medicine, Alexandria University, Alexandria, Egypt), Chiristine Samuel Rezq (Faculty of Medicine, Alexandria University, Alexandria, Egypt), mohamed ibrahim abdo ibrahim (Faculty of Medicine, Alexandria University, Alexandria, Egypt), Marina Samy Ragheb (Faculty of Medicine, Alexandria University, Alexandria, Egypt)

**Bengali**

Ariful Haque (Department of Orthopedic Surgery, Kunming Medical University, Yan an Hospital Affiliated to Kunming Medical University, Kunming, Yunnan, China), Rifath Jahan Antora (Kunming Medical University, Kunming, Yunnan, China)

**Chinese**

Sze Jia Ng (School of Medical Sciences, Universiti Sains Malaysia, Kelantan, Malaysia), Juwie Chuah (School of Medical Sciences, Universiti Sains Malaysia, Kelantan, Malaysia), Yi Liang Lim (School of Medical Sciences, Universiti Sains Malaysia, Kelantan, Malaysia), Yap Siang Jee (School of Medical Sciences, Universiti Sains Malaysia, Kelantan, Malaysia), Li Chuin Chong (School of Data Sciences, Perdana University)

**Filipino**

Ian Christopher Naungayan Rocha (School of Medicine, Centro Escolar University, Manila, Philippines), Kevin Thurston Crispino (Health Emergency Management Bureau, Department of Health, Manila, Philippines)

**French**

Boughalem Younes (Faculty of Medicine and Pharmacy, Cadi Ayyad University, Marrakesh, Morocco)

**German**

Ahmed Hisham Mohamed Hamed (Faculty of Medicine, Cairo University, Cairo, Egypt)

**Hindi**

Venkatesh U (Department of Community Medicine, Vardhaman Mahavir Medical College & Safdarjung Hospital, New Delhi)

**Indonesian**

Graca Jaqueline Vanessa Morena (Faculty of Medicine, Trisakti University - Jakarta, Indonesia & America Evangelical University, Los Angeles CA), Adriana Viola Miranda (Faculty of Medicine, University of Indonesia, Jakarta, Indonesia), Rachel Silency Aritonang (Faculty of Medicine, Trisakti University - Jakarta, Indonesia & Yadika General Hospital, Indonesia)

**Japanese**

Atsuko Imoto (School of Tropical Medicine and Global Health, Nagasaki University, Japan), Dr. Kazumi Kubota (Department of Biostatistics, Yokohama City University, Japan), Koji Aoki (Graduate School of Nursing, Chiba University, Japan)

**Korean**

Ton That Khanh (Faculty of Medicine, School of Medicine and Pharmacy, Da Nang University, Da Nang City, Vietnam)

**Kurdish**

Jeza Muhamad Abdul Aziz (Medical Laboratory Science, College of Health Sciences, University of Human Development, Sulaymaniyah, Kurdistan region-Iraq. Baxshin Research Center, Baxshin Hospital, Sulaymaniyah, Kurdistan Region-Iraq), Mohammed Ibrahim Mohialdeen Gubari (Department of Family and Community Medicine, College of Medicine, University of Sulaimaniyah, Sulaimaniyah, Kurdistan Region, Iraq.), Hemin Fatih Hama Kareem (Department of Psychiatry, Mental Health Center, Baxshin Hospital Sulaymaniyah, Kurdistan Region, Iraq), Rangin Muhamad Hussein (Baxshin Research Center, Baxshin Hospital, Sulaymaniyah-Kurdistan region-Iraq)

**Malay**

Sze Jia Ng (School of Medical Sciences, Universiti Sains Malaysia, Kelantan, Malaysia), Juwie Chuah (School of Medical Sciences, Universiti Sains Malaysia, Kelantan, Malaysia), Yi Liang Lim (School of Medical Sciences, Universiti Sains Malaysia, Kelantan, Malaysia), Yap Siang Jee (School of Medical Sciences, Universiti Sains Malaysia, Kelantan, Malaysia), Li Chuin Chong (School of Medical Sciences, Universiti Sains Malaysia, Kelantan, Malaysia)

**Malayalam**

Venkatesh U (Department of Community Medicine, Vardhaman Mahavir Medical College & Safdarjung Hospital, New Delhi)

**Nepali**

Shyam Prakash Dumre (Central Department of Microbiology, Tribhuvan University, Kathmandu, Nepal; Institute of Tropical Medicine, Nagasaki University, Japan), Kamal Ranabhat (Ministry of Health and Population, Kathmandu, Nepal), Renu Bhandari Dumre (School of Tropical Medicine and Global Health, Nagasaki University, Nagasaki, Japan), Pallavi Koirala (Nepal Health Research Council, Kathmandu, Nepal), Kriti Adhikari (Nepal Health Research Council, Kathmandu, Nepal)

**Pashto**

Irfan Ullah (Kabir Medical College, Gandhara University, Peshawar, Pakistan)

**Portuguese**

Filipa Lucas (Faculdade de Medicina da Universidade de Lisboa)

**Russian**

Mariia Pavlenko (Kyiv City Pediatric Diagnostic Center, Kyiv, Ukraine), Dmytro Pavlenko (Bogomolets National Medical University, Kyiv, Ukraine)

**Spanish**

Ahmad Taysir Atieh Qarawi (Lower Westchester Medical Associates, P.C., Mount Vernon, NY 10550, USA), Joyce Nicole Pineda Ordóñez (Faculty of Medicine, Catholic University of Honduras), José Tomás Ordóñez Aburto (Faculty of Medicine, Universidad de La Frontera, Chile), Andrés Sebastián Estrella Lopez (Faculty of Medicine, Pontificia Universidad Católica del Ecuador), Brianda del Pilar Gómez Olvera (Autonomous University of Mexico State)

**Tamil**

Venkatesh U (Department of Community Medicine, Vardhaman Mahavir Medical College & Safdarjung Hospital, New Delhi)

**Thai**

Nut Koonrungsesomboon (Department of Pharmacology, Faculty of Medicine, Chiang Mai University, Chiang Mai, Thailand), Chidchanok Ruengorn (Department of Pharmaceutical Care, Faculty of Pharmacy, Chiang Mai University, Chiang Mai, Thailand), Surapon Nochaiwong (Department of Pharmaceutical Care, Faculty of Pharmacy, Chiang Mai University, Chiang Mai, Thailand), Penkarn Kanjanarat (Department of Pharmaceutical Care, Faculty of Pharmacy, Chiang Mai University, Chiang Mai, Thailand), Mingkwan Na Takuathung (Department of Pharmacology, Faculty of Medicine, Chiang Mai University, Chiang Mai, Thailand)

**Ukrainian**

Dmytro Pavlenko (Bogomolets National Medical University, Kyiv, Ukraine), Mariia Pavlenko (Kyiv City Pediatric Diagnostic Center, Kyiv, Ukraine), Roman Pavlenko (Bogomolets National Medical University, Kyiv, Ukraine), Iryna Kudlatska-Tyshko (Shupyk National Medical Academy of Postgraduate Education, Kyiv, Ukraine), Ali Dzhemiliev (Shupyk National Medical Academy of Postgraduate Education, Kyiv, Ukraine)

**Urdu**

Somia Iqtadar (King Edward Medical University Pakistan), Usman Ghani (Directorate General Health Services Punjab), Sami Ullah Mumtaz (North Medical Ward, KEMU/Mayo Hospital Lahore), Asad Ali Khan (Khyber Teaching Hospital, Peshawar)

**EMAIL AND ORCID**

ID irida.dajti@gmail.com; 0000-0002-8449-6829

EV enxhivrapi@gmail.com; 0000-0002-2767-5599

JK jolamedicine16@gmail.com; 0000-0002-6893-477X

KSA [kirellossaid98@gmail.com](mailto:kirellossaid98@gmail.com); 0000-0003-0339-9339

FA fatma.ahmed931977@gmail.com; 0000-0001-8456-1603

AES aliaaeffat30@gmail.com; 0000-0002-3828-6038

SFA shehabfathy134@gmail.com; 0000-0002-6964-247X

ZHH zetairon@gmail.com; 0000-0001-6587-8517

MAN mariam.16266094@med.aun.edu.eg; 0000-0002-7937-4229

ES esraajamaleldeeb@gmail.com; 0000-0003-0290-5228

MS moosa.shibani@gmail.com; 0000-0002-4424-2728

HI Helmaismail997@gmail.com; 0000-0003-4329-9519

MAA ameenzabibi@gmail.com; 0000-0003-4050-3051

BS bishersawaf.94@gmail.com; 0000-0001-6751-619X

HAMO hodaomran100@outlook.com; 0000-0003-1840-3599

CSR chiristinesamuel@gmail.com; 0000-0002-7040-4268

miai mohamedibrihem2012@gmail.com; 0000-0002-5216-7545

MSR marinasamy25@gmail.com; 0000-0002-7691-2562

DMAH [arifulhaque58@gmail.com](mailto:arifulhaque58@gmail.com); 0000-0003-4632-5153

RJA [dr.rifathjahan060@gmail.com](mailto:dr.rifathjahan060@gmail.com); 0000-0003-0848-9456

DSJN elaine-1_ng@outlook.com; 0000-0001-5353-6499

DJC juwiechuah@live.com; 0000-0001-6992-3901

DYLL lim.95@hotmail.com; 0000-0001-6152-1959

DYSJ jeeys@hotmail.my

LCC lichuinchong@gmail.com; 0000-0002-3574-1365

ICNR giannisrocha@gmail.com; 0000-0002-8775-6876

KTC kevincrispino16@gmail.com; 0000-0002-6505-2982

BY nsbm2000@gmail.com; 0000-0001-6903-5146

AHMH ahmed.hamed@students.kasralainy.edu.eg; 0000-0002-0843-532X

VU venkatesh2007mbbs@gmail.com; 0000-0001-8059-9364

GJVM [moresoares11@gmail.com](mailto:moresoares11@gmail.com); 0000-0002-3531-0370

AVM [adrianaviola99@gmail.com](mailto:adrianaviola99@gmail.com); 0000-0001-8548-1592

RSA [aritonangrachel@gmail.com](mailto:aritonangrachel@gmail.com); 0000-0003-2702-2691

AI imoto@nagasaki-u.ac.jp; 0000-0003-2744-6160

DKK kubotaa@yokohama-cu.ac.jp

KA koji-aoki@chiba-u.jp

TTK khanhtonthat08@gmail.com; 0000-0003-3462-4244

DJMA [jeza1981@gmail.com](mailto:jeza1981@gmail.com); 0000-0002-1522-1880

MIMG [mohammed.jubar@gmail.com](mailto:mohammed.jubar@gmail.com); 0000-0002-2873-3981

HFHK [dr.hemn@gmail.com](mailto:dr.hemn@gmail.com); 0000-0002-8119-5246

RMH [rangen991@gmail.com](mailto:rangen991@gmail.com); 0000-0001-5938-2224

SPD sp.dumre@gmail.com; 0000-0002-4072-0745

PG prgyawali@nhrc.gov.np

MD meghdhimal2@gmail.com; 0000-0001-7176-7821

KR kamal_raj7@iom.edu.np; 0000-0002-3396-7440

PK pallavik23@gmail.com

KA adhkriti@gmail.com

RBD renu.bdumre@gmail.com; 0000-0001-8039-0309

IU irfanullahecp2@gmail.com; 0000-0003-1100-101X

FL [Filipa-lucas@campus.ul.pt](mailto:Filipa-lucas@campus.ul.pt); 0000-0001-7651-7194

MP kukushkinamd@gmail.com; 0000-0002-9564-6730

DP pavlenkomd@gmail.com; 0000-0001-8761-9559

ATAQ ahmadtqarawi@gmail.com; 0000-0001-7278-9889

JNPO jn.pineda94@gmail.com; 0000-0002-0953-8742

JTOA jtordonezaburto@gmail.com; 0000-0001-8705-4041

ASEL andresestrella823@gmail.com; 0000-0003-1929-7548

BPGO gom-olv@live.com.mx; 0000-0002-5905-8616

NK nkoonrung@gmail.com; 0000-0003-4649-597X

CR chidchanok.r@elearning.cmu.ac.th; 0000-0001-7927-1425

SN surapon.nochaiwong@gmail.com; 0000-0003-1100-7171

PK penkarnk@hotmail.com; 0000-0002-8160-5444

MN k_mingkwan@hotmail.com; 0000-0003-4240-5367

IKT irynakudlatska@gmail.com; 0000-0002-2700-1455

AD [alijemilev@gmail.com](mailto:alijemilev@gmail.com); 0000-0002-0529-7902

RP tjroma1@gmail.com; 0000-0001-8070-5649

SI Somia.iqtadar@gmail.com; h0000-0002-8482-9355

UG usman293eb@gmail.com

SUM drsumumtaz@gmail.com

AAK doctorasadalikhan@gmail.com; 0000-0001-8503-3036
