# Supplementary material for: Perceived Stress of Quarantine and Isolation During COVID-19 Pandemic: A Global Survey
Source: Front Psychiatry. 2021 May 25;12:656664. doi: 10.3389/fpsyt.2021.656664 (PMC8186534; doi:10.3389/fpsyt.2021.656664)
Supplement: Supplementary file 2 [file Data_Sheet_2.docx]

**Appendix-2**

**Perceived stress of quarantine and isolation during COVID-19 pandemic: A Global Survey**

**Running head**: COVID-19 perceived stress of quarantine and isolation

*The TMGH-Global COVID-19 Collaborative†.*

**Supplementary table 1. List of participating countries with recorded response rate**

| ID | Country | Frequency (n) | Percentage (%) |
| --- | --- | --- | --- |
| 1 | Afghanistan | 2 | 0.11 |
| 2 | Albania | 387 | 20.68 |
| 3 | Angola | 4 | 0.21 |
| 4 | Bangladesh | 73 | 3.90 |
| 5 | Belgium | 1 | 0.05 |
| 6 | Bolivia | 4 | 0.21 |
| 7 | Bosnia | 1 | 0.05 |
| 8 | Brazil | 2 | 0.11 |
| 9 | Canada | 2 | 0.11 |
| 10 | Chile | 4 | 0.21 |
| 11 | China | 4 | 0.21 |
| 12 | Czech Republic | 3 | 0.16 |
| 13 | Denmark | 2 | 0.11 |
| 14 | Ecuador | 52 | 2.78 |
| 15 | Egypt | 26 | 1.39 |
| 16 | El Salvador | 1 | 0.05 |
| 17 | France | 25 | 1.34 |
| 18 | Germany | 18 | 0.96 |
| 19 | Greece | 8 | 0.43 |
| 20 | Honduras | 2 | 0.11 |
| 21 | Hungary | 2 | 0.11 |
| 22 | India | 160 | 8.55 |
| 23 | Indonesia | 75 | 4.01 |
| 24 | Italy | 23 | 1.23 |
| 25 | Japan | 12 | 0.64 |
| 26 | Jordan | 4 | 0.21 |
| 27 | Kenya | 1 | 0.05 |
| 28 | Korea | 9 | 0.48 |
| 29 | Kosovo | 2 | 0.11 |
| 30 | Lebanon | 1 | 0.05 |
| 31 | Libya | 39 | 2.08 |
| 32 | Luxembourg | 3 | 0.16 |
| 33 | Malaysia | 13 | 0.69 |
| 34 | Mexico | 55 | 2.94 |
| 35 | Nepal | 126 | 6.73 |
| 36 | Netherlands | 2 | 0.11 |
| 37 | Nigeria | 1 | 0.05 |
| 38 | Oman | 1 | 0.05 |
| 39 | Pakistan | 81 | 4.33 |
| 40 | Palestine | 22 | 1.18 |
| 41 | Peru | 1 | 0.05 |
| 42 | Philippines | 108 | 5.77 |
| 43 | Poland | 2 | 0.11 |
| 44 | Portugal | 21 | 1.12 |
| 45 | Puerto Rico | 1 | 0.05 |
| 46 | Qatar | 4 | 0.21 |
| 47 | Romania | 2 | 0.11 |
| 48 | Russia | 1 | 0.05 |
| 49 | Saudi Arabia | 10 | 0.53 |
| 50 | Scotland | 2 | 0.11 |
| 51 | Seychelles | 1 | 0.05 |
| 52 | Singapore | 1 | 0.05 |
| 53 | Somalia | 1 | 0.05 |
| 54 | South Africa | 1 | 0.05 |
| 55 | Spain | 4 | 0.21 |
| 56 | Sudan | 19 | 1.02 |
| 57 | Thailand | 12 | 0.64 |
| 58 | Timor Leste | 4 | 0.21 |
| 59 | Turkey | 1 | 0.05 |
| 60 | United Arab Emirates | 2 | 0.11 |
| 61 | United Kingdom | 51 | 2.73 |
| 62 | United States | 35 | 1.87 |
| 63 | Vietnam | 317 | 16.94 |
| 64 | NA | 17 | 0.91 |
|  | Total | 1871 | 100.00 |
